# Exceptionally rare *IDH1*-mutant adult medulloblastoma with concurrent *GNAS* mutation revealed by in vivo magnetic resonance spectroscopy and deep sequencing

**DOI:** 10.1186/s40478-023-01531-y

**Published:** 2023-03-20

**Authors:** Roberto Liserre, Francesca Branzoli, Francesca Pagani, Magdalena Gryzik, Manuela Cominelli, Evelina Miele, Małgorzata Marjańska, Francesco Doglietto, Pietro Luigi Poliani

**Affiliations:** 1 Department of Radiology, Neuroradiology Unit, ASST Spedali Civili University Hospital, Brescia, Italy; 2 Paris Brain Institute - Institut du Cerveau (ICM), Centre de NeuroImagerie de Recherche (CENIR), Paris, France; 3 Sorbonne Université, UMR S 1127, Inserm U 1127, CNRS UMR 7225, ICM, F-75013 Paris, France; 4 Pathology Unit, Department of Molecular and Translational Medicine, University of Brescia, P.le Spedali Civili 1, 25125 Brescia, BS Italy; 5 Department of Pediatric Onco-Hematology and Cell and Gene Therapy, Bambino Gesù Children’s Hospital, IRCCS, Rome, Italy; 6 Center for Magnetic Resonance Research, Department of Radiology, University of Minnesota, Minneapolis, MN USA; 7 Fondazione Policlinico Universitario Agostino Gemelli IRCCS, Rome, Italy; 8 Catholic University School of Medicine, Rome, Italy

**Keywords:** Magnetic resonance spectroscopy (MRS), d-2-Hydroxyglutarate (2HG), Isocitrate dehydrogenase 1 and 2 mutation, Medulloblastoma, *GNAS* mutation

## Abstract

Medulloblastoma (MB) is the most common malignant brain tumor occurring in childhood and rarely found in adults. Based on transcriptome profile, MB are currently classified into four major molecular groups reflecting a considerable biological heterogeneity: WNT-activated, SHH-activated, group 3 and group 4. Recently, DNA methylation profiling allowed the identification of additional subgroups within the four major molecular groups associated with different clinic-pathological and molecular features. Isocitrate dehydrogenase-1 and 2 (*IDH1* and *IDH2*) mutations have been described in several tumors, including gliomas, while in MB are rarely reported and not routinely investigated. By means of magnetic resonance spectroscopy (MRS), we unequivocally assessed the presence the oncometabolite D-2-hydroxyglutarate (2HG), a marker of *IDH1* and *IDH2* mutations, in a case of adult MB. Immunophenotypical work-up and methylation profiling assigned the diagnosis of MB, subclass SHH-A, and molecular testing revealed the presence of the non-canonical somatic *IDH1(p.R132C)* mutation and an additional *GNAS* mutation, also rarely described in MB. To the best of our knowledge, this is the first reported case of MB simultaneously harboring both mutations. Of note, tumor exhibited a heterogeneous phenotype with a tumor component displaying glial differentiation, with robust GFAP expression, and a component with conventional MB features and selective presence of *GNAS* mutation, suggesting co-existence of two different major tumor subclones. These findings drew attention to the need for a deeper genetic characterization of MB, in order to get insights into their biology and improve stratification and clinical management of the patients. Moreover, our results underlined the importance of performing MRS for the identification of *IDH* mutations in non-glial tumors. The use of throughput molecular profiling analysis and advanced medical imaging will certainly increase the frequency with which tumor entities with rare molecular alterations will be identified. Whether these findings have any specific therapeutic implications or prognostic relevance requires further investigations.

## Introduction

Medulloblastoma (MB), the most common embryonal brain tumor occurring in childhood, is rarely seen in adults. Increasing evidence indicates that MB occurring in adults may have a different clinical course and harbor additional molecular alterations as compared to their pediatric counterpart [18, 21]. In 2012, an international consensus study [29] has established four major molecular groups with overlapping histopathological features reflecting a considerable biological heterogeneity: Wingless-activated (WNT-MB), Sonic Hedgehog–activated (SHH-MB), group 3 and group 4. Each MB group is associated with well-defined molecular alterations, such as mutations of *PTCH1*, *SUFU* and *SMO* (SHH-MB), *CTNNB1* (WNT-MB) and *MYC* amplification (non-WNT/non-SHH group 3 and 4) [20]. Recent advances in transcriptomic and epigenetic profiling have further refined the molecular classification allowing for the identification of additional subgroups within the major molecular groups, each associated with different clinic-pathological and molecular features [9, 14]. In adults, SHH-MB represents the most frequent molecular group. Here, we report a rare case of MB in a young adult harboring the non-canonical somatic isocitrate dehydrogenase 1 *IDH1(p.R132C)* mutation and an additional concurrent *GNAS* mutation, both rarely described in MB. *IDH1/2* mutations are reported as a mutational cancer driver in several types of tumors, mostly in gliomas [17]. *IDH* mutations in MB are rarely reported and not routinely investigated [20]. Recent improvements of in vivo magnetic resonance spectroscopy (MRS) at 3 tesla (3 T) have allowed detectability of the oncometabolite, D-2-hydroxyglutarate (2HG), a marker of *IDH1/2* mutations [1, 4, 6]. Using both conventional and edited MRS, we unequivocally assessed the presence of high 2HG levels in a non-glial tumor, encouraging pathologist to perform high throughput molecular screenings that confirmed the presence of the *IDH1* mutation and allowed to identify an additional *GNAS* mutation.

## Case presentation

**Fig. 1:**
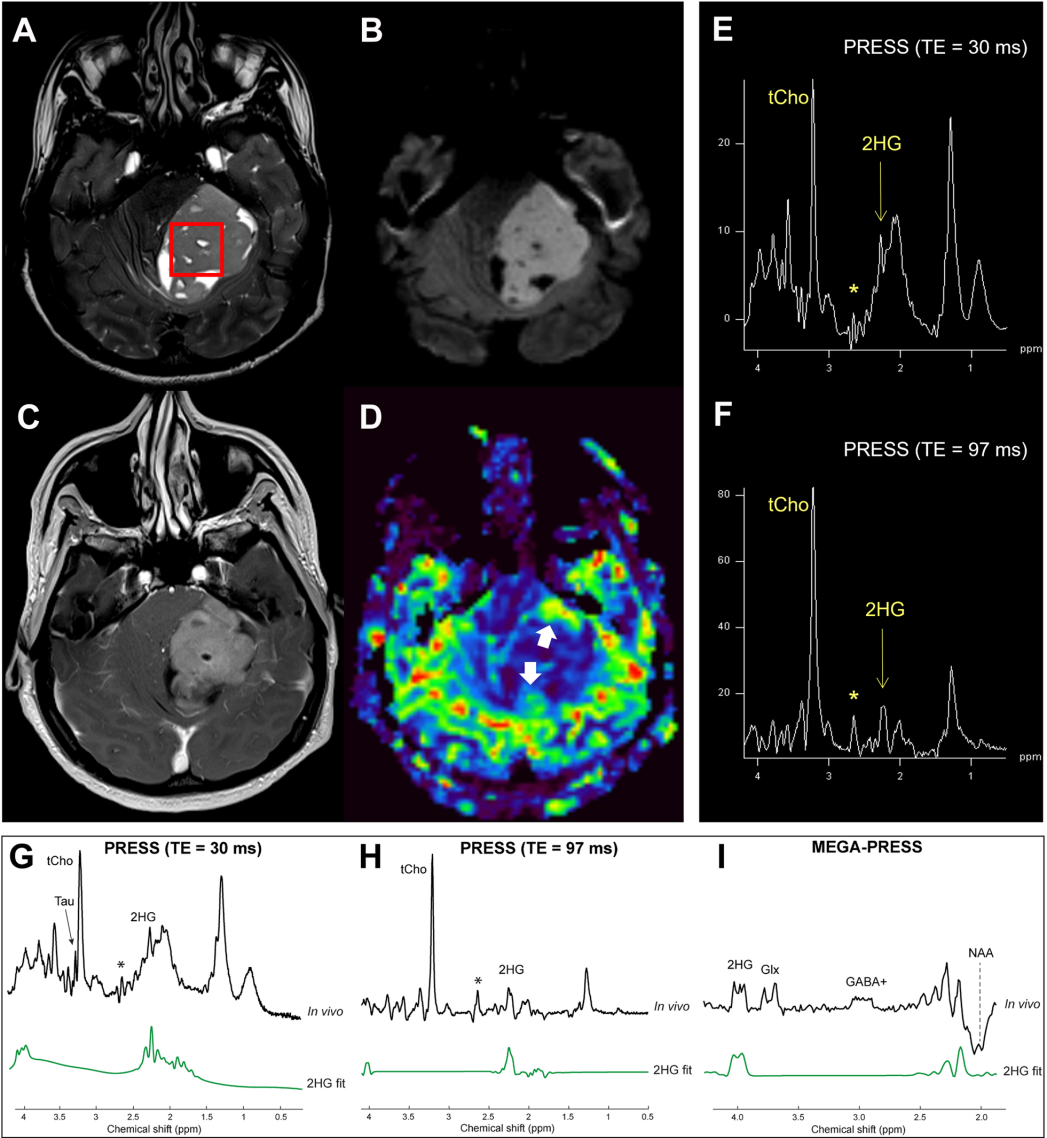
Conventional 3 T MRI (**A**–**D**): large off-midline mixed solid-cystic tumor with sharp margins in the left cerebellar hemisphere showing high signal intensity on T2-weighted images (**A**), homogeneous restricted diffusion of water on diffusion-weighted images (**B**), marked postcontrast enhancement on T1-weighted images (**C**), and few hyperperfused foci on perfusion images (white arrows in **D**). MRS PRESS spectra with TEs of 30 (**E**) and 97 ms (**F**) visualized with a commercial software: a clearly distinct 2HG peak centered at ~ 2.25 ppm can be identified with both methods (yellow, thin arrows). An additional anomalous peak at ~ 2.65 ppm can be seen (asterisks). The MRS voxel placement is shown in (**A**). LCModel analyses for 2HG from conventional PRESS, TE = 30 ms (**G**), optimized PRESS, TE = 97 ms (**H**), and MEGA-PRESS (**I**) spectra. 2HG is reliably quantified in all spectra, as evidenced by very low Cramér-Rao lower bounds (CRLB) (7% for MEGA-PRESS, 4% for optimized PRESS, and 5% for conventional PRESS) and very high 2HG concentrations (16.3 mM from MEGA-PRESS, 14.6 mM from optimized PRESS, and 13.8 mM from conventional PRESS). Tau was detected in both TE = 97 and 30 ms PRESS data (arrow in **G**) (CRLB 9% and 14% respectively; 5 mM and 2 mM, respectively). The additional singlet peak at ~ 2.65 ppm in both PRESS spectra (asterisks in **E**–**H**) was better seen at TE = 97 ms, and tentatively assigned to H-Tau, based on the LCModel fitting (CRLB 9%, 3.2 mM)

**Fig. 2:**
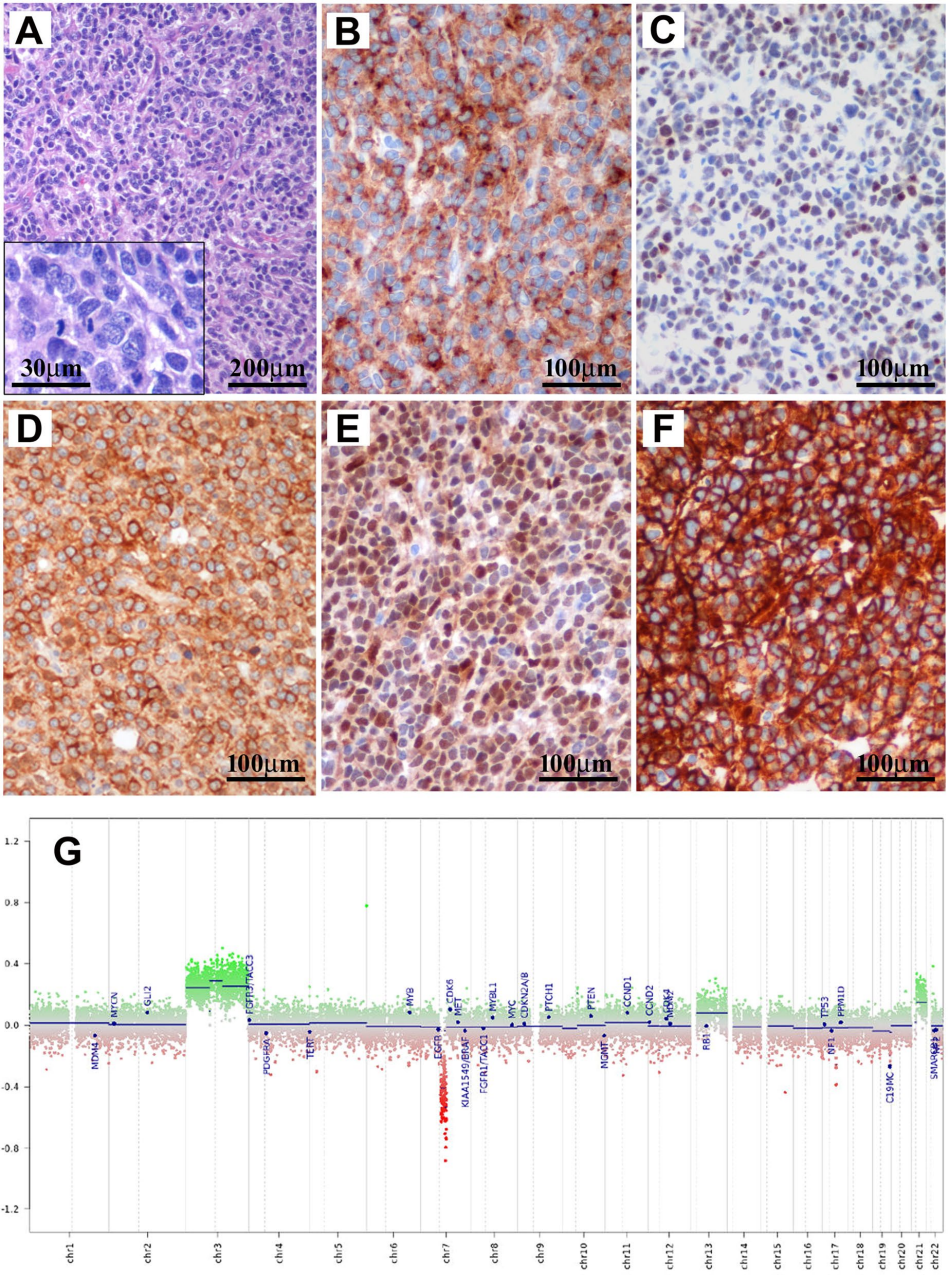
Hematoxylin-eosin staining from a representative tumor area shows densely packed and poorly differentiated neoplastic cells with high nucleus:cytoplasmic ratio and high levels of mitotic activity (**A** and inset). Tumor cells displays immunoreactivity for Synaptophysin (**B**) and low to moderate expression of p53 (**C**). Subgroup-specific immunohistochemical markers show cytoplasmic, but no nuclear β-catenin expression (**D**) as well as diffuse positivity for Yap1 (**E**) and Gab1 (**F**). Overall, these findings are consistent with the diagnosis of SHH MB, p53-wt. The methylation profiling assigned the methylation MB subclass SHH-A (children and adult) according to the v11b4 Brain Tumor Classifier and MB SHH-activated, subtype 4 according to the newest version (v12.5). The copy number profile (**G**) calculated from DNA methylation array data of the tumor sample showing gain of chromosome 3 and a focal loss in chromosome 7 with no other relevant chromosomal aberration

**Fig. 3:**
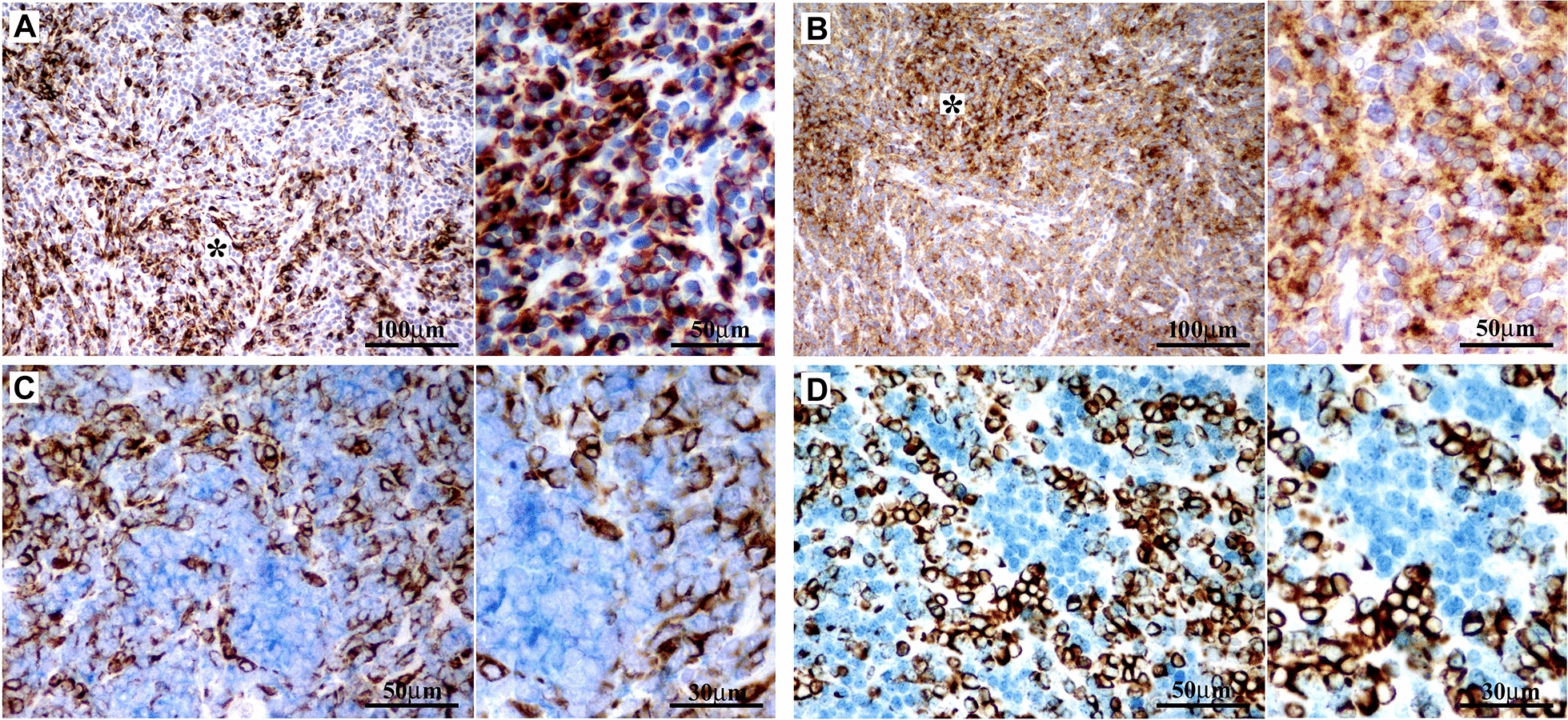
Adjacent sections from a representative tumor area show unexpectedly large areas of strong GFAP immunoreactivity (**A** and inset; asterisk indicate GFAP-enriched areas) along with areas of classical robust Synaptophysin expression (**B** and inset; asterisk indicate Synaptophysin-enriched areas). Double immunostain combining GFAP and Synaptophysin indicates that expression of GFAP (brown, cytoplasmic positivity) and Synaptophysin (blue, cytoplasmic positivity) are mostly mutually exclusive (**C** and inset). Interestingly, double immunostain for GFAP (brown, cytoplasmic positivity) and EBF3 (blue; nuclear positivity), a recognized marker of early neuronal differentiation in medulloblastoma, shows selective EBF3 expression in the GFAP-negative tumor cells (**D** and inset)

**Fig. 4:**
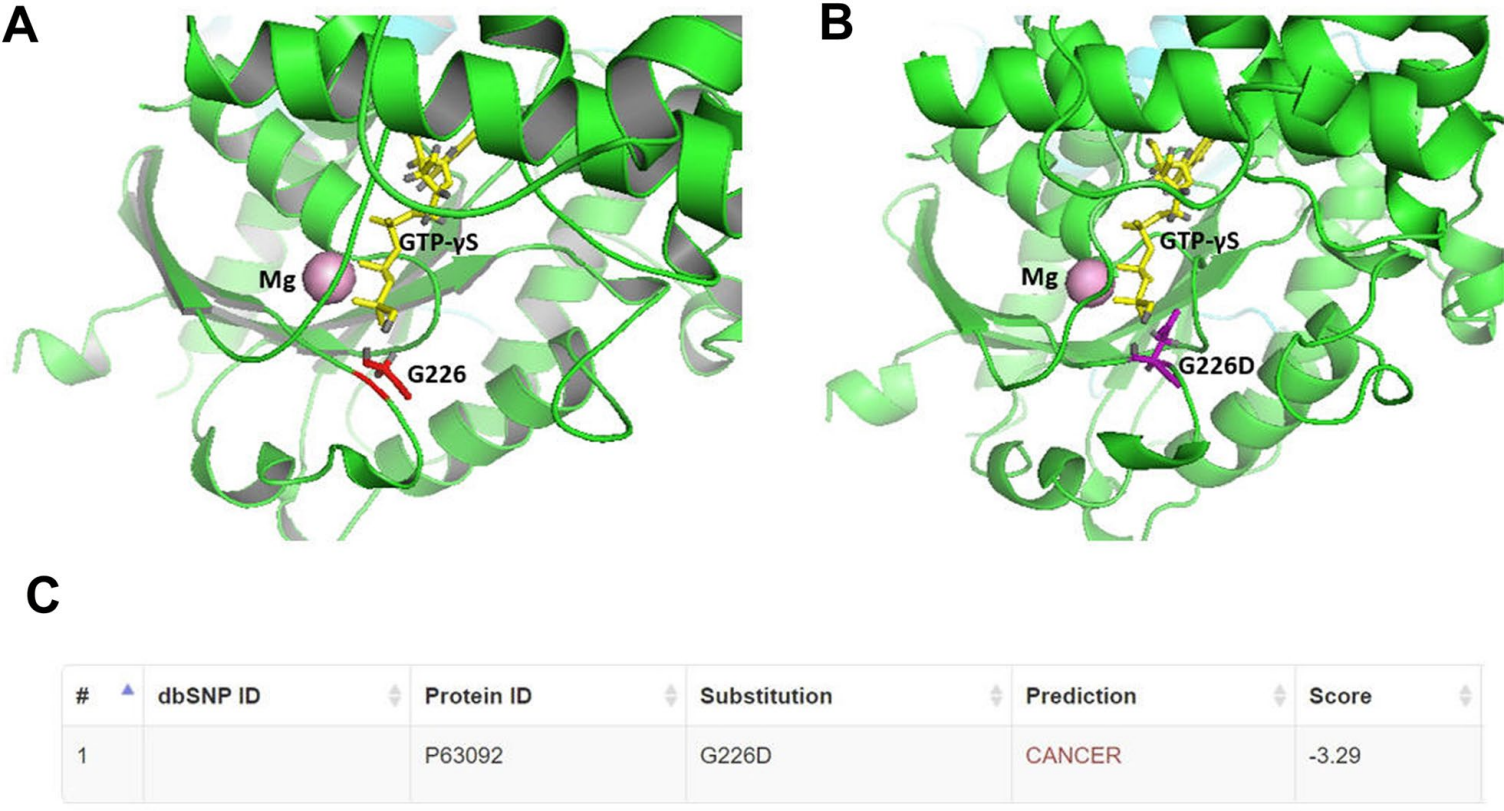
Structure of GTP-γS-bound Gsα protein fragment with indicated amino acid subsitution (**A**, **B**). The structure was generated with PyMol and derived from Sunahara et al. (1997) [28] (PDB: 1AZT). NGS analysis highlighted the presence of a missense mutation (*c.677G > A; p.G226D*) in the *GNAS* gene causing an amino acid change from nonpolar glycine (*G226*) to negatively charged aspartic acid (**D**) that may affect Gsα protein conformation and function. Moreover, *G226* is in the Switch II region, one of the two loops undergoing structural changes upon GTP binding essential for binding and activation of adenylyl cyclase [8]. The G3 box (DXXG), that overlaps this region, is involved in binding a Mg^2+^ through *Asp223* and, more importantly, in a hydrogen bonding with GTP through *Gly226* [22]. Thus, Gsα *G226D* mutant could be likely present in an inactive (GDP-bound) state and, due to the disruption of a hydrogen bond network, unable to bind GTP. Prediction of clinical significance of *G226D* mutation (**C**). The prediction obtained using the Functional Analysis through Hidden Markov Models v2.3 tool [26], indicated the *G226D* mutation as a potentially cancer-associated alteration, showing a high probability of the prediction with a score − 3.29 (cutoff: −0.75)

A 26-year-old male presented with cerebellar syndrome characterized by headache, vomiting, posture unsteadiness and nystagmus. Brain computed tomography (CT) scan showed a slightly hyperdense large lobulated space-occupying left cerebellar hemisphere lesion causing decompensated hydrocephalus. Conventional 3 T MRI (MAGNETOM-Skyra scanner; Siemens Healthcare) confirmed the presence of a large cerebellar off-midline mixed solid-cystic tumor with sharp margins, showing high signal intensity on T_2_-weighted images and marked post-contrast enhancement on T_1_-weighted images, with homogeneous restricted diffusion consistent with high cellularity and a few hyperperfused foci. These findings suggested, all together, the diagnosis of a SHH-MB (Fig. 1A-D). For the MRS acquisition, pre-contrast T_2_-weighted TSE images were used to position a cubic 2.5 × 2.5 × 2.5 cm^3^ (15.625 ml) spectroscopic volume-of-interest (VOI) (Fig. 1A). MR spectra were acquired using conventional PRESS (echo-time (TE) = 30 ms) as well as 2HG-optimized PRESS (TE = 97 ms) [7] and spectral editing MEGA-PRESS [4, 7] sequences. Surprisingly, a distinct peak at ~ 2.25 ppm suggestive of 2HG was detectable in the conventional short-TE MR spectrum (Fig. 1E, G). This finding was confirmed with the two additional MRS acquisitions customized for 2HG detection [4], which revealed unusual very high 2HG concentration (Fig. 1F, H, I). Also, taurine (Tau) and an additional peak at ~ 2.65 ppm tentatively assigned to hypotaurine (H-Tau) according to LCModel fitting [24] were observed (Fig. 1E–H). Cerebrospinal fluid examinations and brain/spine contrast-enhanced MRI revealed no evidence of dissemination. Patient underwent gross total resection followed by conventional radiotherapy treatment. Because of significant radiation-induced bone marrow suppression, no adjuvant chemotherapy treatment was advised. At 15 months follow-up, patient condition was stable with no MRI-visible recurrence. Neuropathological examination revealed a classic MB histology composed of small to medium sized primitive cells with no anaplastic or large-cell features and high mitotic activity (Fig. 2A) without evidence of a desmoplastic micronodular architecture and negative reticulum staining (not shown). Tumor cells were positive for synaptophysin and showed low-to-moderate immunoreactivity for p53 (Fig. 2B–C). INI-1 and ATRX expression were preserved and the Ki67 proliferative index raised up to 30% (not shown). Immunohistochemical sub-classification performed according to the consensus panel [15] showed immunoreactivity for GAB1, YAP1 and cytoplasmic, but not nuclear positivity for β-Catenin (Fig. 2D-F). Overall, data were consistent with SHH-MB, *TP53* wild-type. Methylation profiling was performed on DNA extracted from FFPE tissue section in enriched tumor areas (tumor purity > 90%) and processed using Infinium Methylation EPIC BeadChip (850k) array (Illumina). Methylation-based tumor classification using the methylation classifier v11b4 (available at https://www.molecularneuropathology.org) assigned the methylation class MB, subclass SHH-A (children and adult) with a calibrated score of 0.92 [5]. The reanalysis of the samples with the most recent version of the methylation classifier (v12.5) assigned them to the same methylation class (MB SHH-activated, subtype 4) with a calibrated score of 0.88. Methylation data have been deposited in NCBI’s Gene Expression Omnibus (GEO; Series accession number GSE225302) and are accessible through https://www.ncbi.nlm.nih.gov/geo/query/acc.cgi?acc=GSE225302. High-density DNA methylation arrays allowed for determining copy number alterations that were consistent with gain of chromosome 3 and focal loss in chromosome 7 with no other relevant chromosomal aberration such as *MYCN* or *MYC* amplification and/or deletions of chromosome 9q (*PTCH1*) (Fig. 2G). Of note, gain of chromosome 3 may be relevant as chromosome 3q is a frequent cytogenetic alteration of MB SHH-A methylation class occurring in adults. Unexpectedly, immunostainings highlighted tumor components displaying a robust and diffuse GFAP immunoreactivity, usually not present in MB, along with tumor areas displaying a classical immunoreactivity for Synaptophysin (Fig. 3A, B). Interestingly, double immunostains combining GFAP and Synaptophysin indicated that, in the GFAP-enriched areas, expression of GFAP and Synaptophysin was mostly mutually exclusive (Fig. 3C). We have previously reported that Early B-cell factor 3 (EBF3) is highly expressed in MB, promotes neuronal differentiation in early undifferentiated progenitor cells and may be considered a marker of early neuronal differentiation [10]. As expected, double immunostain combining GFAP and EBF3 showed a selective EBF3 expression in the GFAP-negative MB tumor component (Fig. 3D), suggesting co-existence of two different major tumor subclones with either glial or early neuronal differentiation. MRS findings and the peculiar immunophenotype encouraged us to perform additional molecular analysis, including investigation of *IDH1/2* status, not routinely assayed in MB. Immunostaining using the specific monoclonal antibody recognizing the missense *IDH1(p.R132H)* mutation, detected in more than 90% of *IDH*-mutated gliomas, provided negative result (data not shown). We therefore performed the pyrosequencing assay (PyroMark system using “*IDH1/2* status” kit for Qiagen-Diatech) that revealed the rare *IDH1(p.R132C)* mutation, confirming the MRS findings. Interestingly, in addition to the *IDH1(p.R132C)* mutation, NGS analysis performed on Illumina MiSeq using Myriapod® NGS-IL56G Onco-panel (NG032, Diatech-Pharmacogenetics) highlighted a concurrent high-frequency missense mutation (*c.677G > A*; p.G226D) in the *GNAS* gene producing an amino acid change from nonpolar glycine (G226) to negatively charged aspartic acid that may affect Gsα protein conformation and function (Fig. 4A-B). This mutation is not cataloged as a variant with clinical significance in any available database (NCBI, ClinVar, The Cancer Genome Atlas, cBioPortal, COSMIC). However, the possible molecular changes that could affect the GTP binding capacity suggest a pathogenic significance. Accordingly, the prediction obtained using the Functional Analysis through Hidden Markov Models v2.3 tool [26], indicated the G226D mutation as a potentially cancer-associated alteration, showing a high probability of the prediction with a score − 3.29 (cutoff: -0.75) (Fig. 4A–C). By microdissection, we also performed NGS analysis on the separated GFAP-enriched and GFAP negative MB components. Of note, while the *IDH1(p.R132C)* mutation has been found in both components, albeit at different allele fractions (26% vs. 12.4%; GFAP-enriched and GFAP negative MB components, respectively), *GNAS* mutation has been found only in the GFAP-negative component. The molecular work up did not reveal any additional molecular alteration usually seen in *IDH*-mutant gliomas.

## Discussion and conclusions

*IDH1/2* mutations lead to neo-enzymatic activity resulting in reduction of α-ketoglutarate to 2HG [25]. Accumulation of 2HG plays a crucial role in promoting oncogenesis by altering cellular metabolism, epigenetic regulation and redox homoeostasis [16]. It has been hypothesized that different *IDH* mutations have different prognostic values due to variable genome-wide DNA-methylation levels [30]. *IDH* mutations in CNS are frequent in gliomas where the “canonical” *IDH1(p.R132H)* represents more than 90% of the mutations. *IDH1(p.R132H)* has relatively low 2HG production capacity, while other “non-canonical” *IDH* mutations, such as *IDH1(p.R132C)* (rare in gliomas, prevalent in other tumors), have about ten-fold lower Michaelis constant with more affinity with substrate and higher neo-enzymatic efficiency, accounting for higher 2HG concentrations [25, 30]. Not surprisingly, in our case 2HG concentration assessed with MRS was much higher compared to typically reported values for *IDH1*-mutated gliomas [4, 7]. Moreover, 2HG concentrations were previously found to positively correlate with tumor cellularity [7] and depend on type of *IDH1/2* mutations [30]. Consistently, our SHH-MB harbored *IDH1(p.R132C)* mutation and had high cellularity, a key feature of MB. Analysis of non-*IDH1(p.R132H)* mutations is hampered by the fact that the highly reliable antibodies are only available for the most common *IDH1(p.R132H)* mutation and *IDH1/2* molecular analysis is necessary for diagnosis. 2HG detection by MRS may contribute to the identification of *IDH1/2* mutations [19], particularly in peculiar cases, as hereby reported. Only two previous reports described *IDH1* mutations in MB, both SHH-activated: an *IDH1(p.R132S)* mutation in a middle-aged woman [27] and an *IDH1(p.R132C)* mutation in an early adolescent [12]. However, in these reports, there were no MRS findings suggesting the presence of *IDH1/2* mutations, which were an incidental finding following the deep sequencing. We are aware that the MR features, including MRS markers, are compatible or supportive of specific abnormalities, but lack the specificity to be actually diagnostic, and definitive diagnosis requires sequencing confirmation from tissue. Indeed, the presence of an *IDH* mutation may be found also in patients with L-2-hydroxyglutaric aciduria, a rare autosomal recessive condition affecting exclusively the central nervous system with a massive increase of 2HG possibly detectable by MRS. However, our patient did not show any neurological symptoms related to the disease (such as psychomotor retardation, cerebellar ataxia and variable macrocephaly or epilepsy) nor typical MRI findings including varying degrees of subcortical leukoencephalopathy and cerebellar atrophy. In addition, MRS data analyzed in normal appearing brain regions far away from the cerebellar lesion did not show any evidence of 2-HG accumulation. Thus, the considerable level of 2HG detected by MRS in our patient was highly suggestive of a *IDH1/2* mutated tumor. In our case, other MRS findings (Tau detection, very low levels of *N*-acetyl-aspartate and total creatine and very high levels of lipids and total choline), were also consistent with diagnosis of SHH-MB [3, 23]. Conversely, H-Tau has been rarely reported so far in brain tumors analyzed by ex vivo MRS [2], and it is a poorly understood metabolite whose function has yet to be elucidated. Overall, our report provides new insights into the utility of MRS in brain tumors. Non-invasive in vivo MRS in adult and pediatric brain tumors can be extremely useful as it may contribute to individuate novel tumor features that motivate further molecular analysis and may help to identify novel peculiar molecular alterations. As such, in our case NGS analysis highlighted a concurrent *GNAS* missense mutation (*c.677G > A*). In tumors, mutations of Gsα lead to dysregulation of cAMP cellular level that may be responsible for oncogenic transformation. There are few reports describing *GNAS* mutations in MB [31]. It is reported that decreased expression of GNAS in MB correlates with tumor aggressiveness [13]. Of note, low levels of *GNAS* transcripts and inhibition of Gsα GTPase function activate SHH signaling and define a subset of aggressive SHH-MB, highlighting *GNAS* mutation as a potential prognostic biomarker for treatment stratification of SHH-MB. However, how this mutation may contribute to the MB development deserve to be further investigated. Interestingly, in our case *GNAS* mutation has been selectively found within the GFAP-negative component raising the question if this patient may have a rare case of a mixed MB and glioma. However, the *IDH1(p.R132C)* mutation has been found in both GFAP-enriched and GFAP-negative components, albeit with higher allelic frequency within the latter, suggesting a common origin of the two components. A cell lineage study of the tumor specimen and/or a spatial single cell analysis would add additional information about the possible tumor evolution, clarifying if this is a rare case of tumor growth due to oncogenic *IDH1* and *GNAS* mutations that both occurred early in the tumor or if the *IDH1* mutation was a later event branching the GFAP-enriched component towards a glial phenotype. To our knowledge, this is the first report of 2HG non-invasive detection in a non-glial tumor that contributed to reveal a rare *IDH1(p.R132C)* mutation associated with adult-onset SHH-MB. In addition, our case represents the first comparison between a conventional and two customized MRS acquisitions for 2HG identification. Interestingly, our data indicated that 2HG assessment may be also possible with routine MRS sequences, in case of very high 2HG concentration. Future dissemination of specific MRS expertise should favor incorporation of customized MR sequences in the clinical practice [11]. This report emphasizes the importance of performing these investigations also in non-glial tumors that may help to unmask rare molecular alterations of potential value for tumor stratification and patient management. Further studies will be necessary to establish the effective frequency of *IDH* and *GNAS* mutations in MB and their prognostic relevance in the way of personalized medicine. *IDH1*-mutated SHH-MB may represent an underestimated specific subgroup with distinctive molecular profile leading to tumor development from adolescence to adulthood.

## Data Availability

All data generated or analyzed during this study are included in this published article.

## Abbreviations

*IDH*: Isocitrate dehydrogenase
GFAP: Glial fibrillary acidic protein
MRI: Magnetic resonance imaging
MRS: Magnetic resonance spectroscopy
